# The Interactions of Quantum Dot-Labeled Silk Fibroin Micro/Nanoparticles with Cells

**DOI:** 10.3390/ma13153372

**Published:** 2020-07-30

**Authors:** Longxing Niu, Meijing Shi, Yanfei Feng, Xiaoxiao Sun, Ying Wang, Zhiling Cheng, Mingzhong Li

**Affiliations:** National Engineering Laboratory for Modern Silk, College of Textile and Clothing Engineering, Soochow University, No. 199 Ren’ai Road, Industrial Park, Suzhou 215123, China; niu18860902006@163.com (L.N.); meijing627@163.com (M.S.); fengyanfei0123@126.com (Y.F.); 20185215041@stu.suda.edu.cn (X.S.); wangying9518@163.com (Y.W.); C1473537105@163.com (Z.C.)

**Keywords:** silk fibroin, micro/nanoparticles, quantum dots, adhesion, internalization

## Abstract

When silk fibroin particles are used for controlled drug delivery, particle size plays a key role in the location of the carrier on the cells as well as the transport pathway, utilization efficiency, and therapeutic effect of the drugs. In this study, the interactions of different-sized silk fibroin particles and cell lines were investigated. Silk fibroin microparticles with dry size of 1.9 ± 0.4 μm (2.7 ± 0.3 μm in wet state) and silk fibroin nanoparticles with dry size of 51.5 ± 11.0 nm (174.8 ± 12.5 nm in wet state) were prepared by salting-out method and high-voltage electrospray method, respectively. CdSe/ZnS quantum dots were coupled to the surface of the micro/nanoparticles. Photostability observations indicated that the fluorescence stability of the quantum dots was much higher than that of fluorescein isothiocyanate. In vitro, microparticles and nanoparticles were co-cultured with human umbilical vein endothelial cells EA.hy 926 and cervical cancer cells HeLa, respectively. The fluorescence test and cell viability showed that the EA.hy926 cells tended to be adhered to the microparticle surfaces and the cell proliferation was significantly promoted, while the nanoparticles were more likely to be internalized in HeLa cells and the cell proliferation was notably inhibited. Our findings might provide useful information concerning effective drug delivery that microparticles may be preferred if the drugs need to be delivered to normal cell surface, while nanoparticles may be preferred if the drugs need to be transmitted in tumor cells.

## 1. Introduction

A drug delivery system consists of a drug carrier in which the active drug is dissolved, dispersed, or encapsulated, or onto which the active ingredient is adsorbed or attached [1]. Diverse drug carriers, including nanoparticles, microspheres, microcapsules, pills and emulsions have been developed to meet the different environments and requirements of the drugs, such as the route of administration, the site of action, the rate of release, and the mode of action [2,3]. Micro/nanoparticles are clever drug carriers due to their unique characteristics, such as small size and large surface area, in addition to their ability to cross the blood–brain barrier, enter the pulmonary system, and be absorbed through the tight junctions of endothelial cells of the skin [4,5]. When micro/nanoparticles are used as drug carriers, the challenge is to deliver the drugs to a specific release site, which is closely related to the interactions between particles and cells.

When drug-loaded micro/nanoparticles act on target cells, they initially adhere to the cell membranes [6]. Cell membranes as barriers tend to be impermeable to many large particles, causing particles to be concentrated in cells or excluded from cells [7]. Micro/nanoparticles attached to the surface of the cell membranes can release the drug extracellularly, and the drugs influence the cellular signaling components (receptors, signal activators, transducers) in membrane microdomains or are ingested by endocytosis [8]. However, some drugs are easily degraded by various digestive enzyme systems in lysosomes, and some drugs play a pharmacological role in specific organelles, such as therapeutic nucleic acids acting in the cell cytoplasm or nucleus [9,10]. Therefore, it is necessary for carriers to deliver drugs to the specified organelles. Through an energy-dependent pathway, drug-loaded particles can be internalized by the cells, and drugs can be released intracellularly [6]. This process is described as follows: the particles form membrane vesicles known as early endosomes, the early endosomes mature into late endosomes and then lysosomes [11], the release of cytotoxic drugs is triggered by the pH of the lysosome [12]. Particles escaping from lysosomes are dispersed in the cytoplasm to release drugs, or to be transported into mitochondria where they exert unique pharmacological activities such as ATP depletion in multidrug resistant cancer cells. Nanoparticles modified by molding peptides could even enter the cell nucleus to release drugs [13]. Therefore, the carrier/drug complex enters the cell or does not directly determine the cellular site of the drugs, the intracellular transport pathway of the drugs, the efficiency of the drugs, or the effect of drugs on the cells, which is an important factor that must be considered when designing the corresponding carrier based on the different mechanisms of drugs.

It is notable that the presence of particles inside or outside the cell is related to the particle size [14]. The size of the unite cell is a few to tens of microns [15], and they were adhered to the surface of microspheres with a size of hundreds of microns [16]. Nanoparticles are smaller than cells and can be internalized by different ingestion mechanisms. The size of vesicles involved in clathrin-mediated endocytosis is approximately 100–200 nm, while the size involved in caveolae-mediated endocytosis is approximately 50–80 nm [17,18], and the size involved in phagocytosis is more than 250 nm [19]. The final destination of the internalized particles in the cells is also determined by the particle diameter, as Fröhlich E reported that nanoparticles 5 nm in diameter were taken up into the nucleus of human fibroblasts, while particles larger than 30 nm were retained in the cytoplasm [20]. Therefore, the adhesion/uptake behavior between cells and particles, the internalization pathway of particles, and the localization of particles in cells are related to the particle size, which will affect the release of drugs at specific locations. Particles of different sizes should be designed as carriers according to the mechanism of different drugs acting on cells. However, carrier delivery of drugs to target cancer cells should reduce toxic side effects on normal cells. Compared to normal cells, cancer cells have been shown to express different amounts of receptors on their surface, which may affect the available binding sites of cargos and their uptake [21,22]. Therefore, the cell type (e.g., phagocytic vs. nonphagocytic, cancer vs. normal cells, and monocytes vs. macrophages) of action should also be considered when designing the carrier size. If the biological behavior of different-types of cells to different-sized particles can be clarified, it can provide an important basis for the selection of carrier size. An appropriate technical method is required to clarify the dynamic cellular process.

Fluorescence labeling is a significant tool for visualizing cellular structure and studying dynamic cellular process in living cells [23]. Despite the major advantages of organic fluorophores, their applications are also limited by narrow excitation bands, broad emission, fast photobleaching, and weak fluorescent emission [24]. Quantum dots (QDs) are fluorescent semiconductor nanocrystals that have attracted the attention of researchers due to their adjustable size emission, high brightness, and excellent photostability [23,25]. When applied as a biological tracer, QD tracking can be readily used on living cells to decipher complex cellular events such as cell membrane dynamism, signal transduction, or intracellular transport [26]. However, QDs synthesized in organic solvents have hydrophobic surfaces and cannot be applied in aqueous buffers [27]. Therefore, these particles are usually modified by hydrophilic groups such as carboxyl (–COOH), amino (–NH) and thiol (–SH) groups [28]. Modified quantum dots can be stably labeled with a target object by ligand exchange, chemical coupling, and electrostatic interactions [29]. Under the mediation of 1-Ethyl-3-(3-dimethylaminopropyl) carbodiimide hydrochloride (EDC) and N-hydroxysuccinimide (NHS), quantum dots were shown to label the surface of angiogenin because the carboxyl groups of the quantum dots were conjugated with the amino groups of angiogenin, indicating that the quantum dots could be labeled on the surface of biological molecules containing amino groups [23].

Silk fibroin from *Bombyx mori*, is a versatile material for drug delivery systems because of its unique combination of biocompatibility, controllable biodegradability, mechanical stability, and diverse processing windows [30]. The active amino groups of silk fibroin provide opportunities for chemical modification [31], and it is feasible to label this protein with quantum dots. As a drug carrier, silk fibroin can be processed as films, gels, nanoparticles, microspheres, and 3D-scaffolds [32]. The structural hierarchy, composition, and crystallinity of silk can be modified to achieve the designed release behavior without compromising the drug activity [33,34]. Different kinds of drugs such as genes, biological drugs, and small molecules exhibit controlled release from silk carries because silk stabilizes drugs via entrapment, adsorption, and encapsulation [35,36].

In our previous study, we prepared silk fibroin microparticles by high-voltage electrospray. Normal cell, mouse fibroblast cells L-929 were adhered to the surface of the microspheres, and cell proliferation was promoted [16,37]. However, the interactions between different cell lines and different-sized silk fibroin particles are not clear. The purpose of this paper is to label silk fibroin particles with quantum dots to clarify the biological behavior of different cells toward micron-sized and nanometer-sized silk fibroin particles. We hypothesized that quantum dots labeled-silk fibroin particles have stable fluorescence intensity, and the cells were adhered to the surface of silk fibroin microparticles but internalized the silk fibroin nanoparticles. Based on this hypothesis, silk fibroin microparticles (SFMPs) and silk fibroin nanoparticles (SFNPs) were prepared by salting-out method and high-voltage electrospray method, respectively, and the size and morphology of the particles were observed by scanning electron microscopy. The –COOH groups of QDs were activated by EDC and coupled with the amino groups of silk fibroin to form QD-SFMPs and QD-SFNPs. The products were characterized by Fourier transform infrared (FTIR) spectroscopy and fluorescence spectroscopy. The fluorescence stability derived from QDs was investigated and compared with FITC. Human umbilical vein endothelial cells EA.hy926 and cervical cancer cells HeLa were co-cultured with silk fibroin particles of different sizes, respectively. The effect of the particles on cell proliferation was investigated by a cell counting kit (CCK-8) and the adhesion/internalization behaviors of different-types of cells to different-sized silk fibroin particles were observed by inverted fluorescence microscope.

## 2. Materials and Methods

### 2.1. Preparation of Silk Fibroin Solution

*Bombyx mori* raw silks were degummed following a previously described procedure [38]. Briefly, silk fibers (Nantong, China) were boiled three times in 0.5 mg/mL Na_2_CO_3_ aqueous solution for 30 min to remove sericin and dried at 60 °C after thorough rinsing with distilled water for subsequent experiments. The 10 g extracted fibers were dissolved in 100 mL 9.3 mol/L LiBr solution at 60 ± 2 °C for 1 h. After being completely cooled, the regenerated silk fibroin solution was obtained after dialysis (MWCO, 8–14 kDa) in deionized water at 4 °C for 3 days. The resulting silk fibroin solution was centrifuged (Heraeus PICO17, Thermo Scientific Company, Darmstadt, Germany) at 10,000 rpm for 5 min to remove aggregates and undissolved impurities. The concentration of resulting solution was ~40 mg/mL. Then the solution was stored in a 4 °C refrigerator before use.

### 2.2. Preparation of Silk Fibroin Micro/nanoparticles

SFMPs were prepared by inducing phase separation from an aqueous silk fibroin solution by the addition of a potassium phosphate solution. First, the concentration of KH_2_PO_4_ and K_2_HPO_4_ solution was 1.25 mol/L, and then, the KH_2_PO_4_ solution was adjusted to pH = 8 with K_2_HPO_4_ solution. The obtained potassium phosphate solution was mixed with 5 mg/mL silk fibroin solution in a volumetric ratio of 5:1. The mixed solution was placed at 4 °C refrigerator for 2 h after mixing evenly, and then placed at room temperature for 12 h. The dispersion of microparticles was centrifuged at 5000 rpm for 15 min. Subsequently, microparticles were re-dispersed in purified water and washed three times. The final microsphere solution was stored at 4 °C before use. 

**Figure 1:**
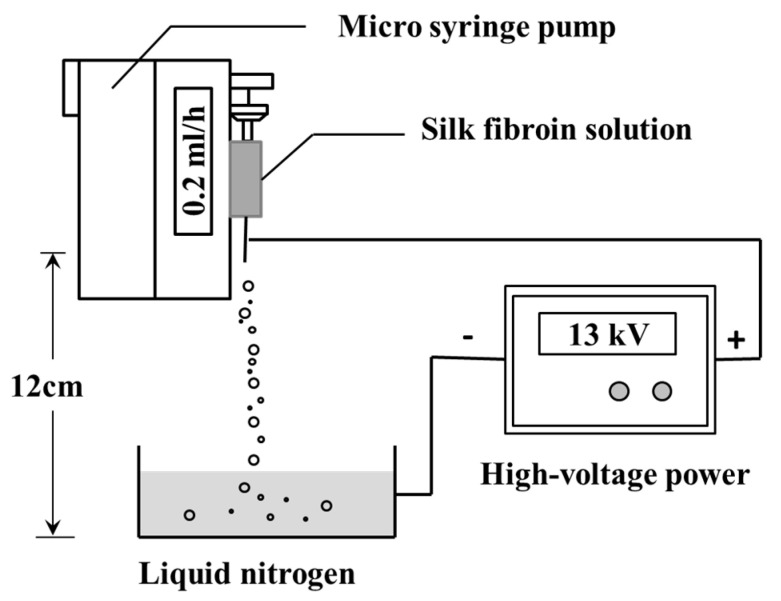
Schematic presentation of the preparation of silk fibroin nanoparticles (SFNPs) by high-voltage electrospray.

SFNPs were prepared by using a high-voltage electrostatic generator (DW-P503-4ACCD, Dongwen High Voltage Power Plant, Tianjin, China) and a micro-injection pump (WZS50F2, Zhejiang University Medical Instrument Co., Ltd., Hangzhou, China). A nozzle with a diameter of 0.5 mm was connected to the syringe, and the entire assembly was fixed on the pump. The distance between the needle and the collection box was fixed at 12 cm and the electrostatic voltage was 13 kV. The flow rate was fixed at 0.2 mL/h. Then, 60 mg of glycerol was added to the 10 mL of silk fibroin solution with a concentration of 20 mg/mL. The mixed solution was injected into the syringe. In the high-voltage electrostatic field, the electrostatic stress caused the solution at the needle tip to break into droplets, and the resulting droplets were continuously collected and frozen in a liquid nitrogen bath (Figure 1). Subsequently, the frozen droplets were freeze-dried in Virtis Genesis 25-LE lyophilizer (Virtis, Gardiner, NY, USA) for 48 h and suspended in deionized water. The upper solution was centrifuged at 13,000 rpm for 20 min to separate the SFNPs.

### 2.3. Preparation of Fluorescence Labeled Silk Fibroin Micro/Nanoparticles

First, 100 mg of FITC (Sigma, molecular weight 398.4) in dimethyl sulfoxide was slowly added to 10 mL of 1 mg/mL silk microparticle suspension. The reaction was allowed to proceed for 12 h at room temperature in the dark. To remove the unconjugated FITC, the precipitate was repeatedly washed and centrifuged. The solution was dialyzed in phosphate buffer saline (PBS, 10 mM, pH = 7.4) for 3 days and changed every 2 h to obtain fluorescence labeled silk fibroin microparticles, which were called FITC-SFMPs.

The 10 μL CdSe/ZnS QDs synthesized by Wuhan Jiayuan Quantum Dot Co., Ltd (Wuhan, China), were incubated with 1-ethyl-3-(3-dimethylaminopropyl) carbodiimide hydrochloride (EDC, Sigma-Aldrich, St. Louis, MO, USA) and N-hydroxysuccinimide (NHS, Sigma-Aldrich, St. Louis, MO, USA) in 1 mL PBS buffer for 1 h at 4 °C (EDC/NHS = 2:1 in molar ratio). Then, 2 mL of 1 mg/mL SFMPs were added and the pH was adjusted to 7.4 with 0.1 M 2-(N-morpholino) ethanesulfonic acid (MES, Sigma-Aldrich, St. Louis, MO, USA). After the mixed solution was incubated at 4 °C for 4 h, it was collected in dialysis tubes (MWCO 8–14 kDa) and dialyzed in PBS for 3 days to remove unreacted excess EDC, NHS and QDs. The dialyzed solution was centrifuged at 28,000 rpm for 30 min, and the obtained precipitates were re-dispersed in PBS and washed three times. Finally, QD-SFMPs were collected and stored at 4 °C. The method to prepare QD-SFNPs is the same as QD-SFMPs.

### 2.4. Scanning Electron Microscopy

The surface morphology of the SFMPs and SFNPs was assessed by scanning electron microscopy (SEM, S-4800, Hitachi, Tokyo, Japan). The SFMPs and SFNPs solution (1 mg/mL) were used for SEM analysis after ultrasonication, and the suspensions were dropped on silicon pellet and dried in an oven at 60 °C. The samples were sputter-coated with gold for 90 s under vacuum. All particle sizes of the SFMPs and SFNPs were analyzed using SEM images with Nano Measurer analysis software (Department of Chemistry in Fudan University, Shanghai, China), for each particle, the average equivalent circular diameter was determined by a total of 100 particles.

### 2.5. Dynamic Light Scattering

The average particle size of silk fibroin micro/nanoparticles was determined by dynamic light scattering (DLS) using a Nano-ZS90 (Malvern Instruments, Malvern, UK). Size was measured in automatic mode for the mean value of three test run repetitions.

### 2.6. Fourier Transform Infrared Spectroscopy

The FTIR spectra of the fluorescence-labeled silk fibroin particles were obtained using a Nicolet 5700 FT-IR spectrometer (Nicolet Instrument, Thermo Company, Madison, WI, USA). All spectra were taken in the spectral region of 400–4000 cm^−1^ using an accumulation of 64 scans and a resolution of 4.0 cm^−1^.

### 2.7. Fluorescence Spectroscopy

All samples were suspended in PBS (1 mg/mL). Fluorescence spectra between 590 and 700 nm (excitation, 550 nm) were recorded at room temperature on a Horiba Fluoromax-4 fluorescence spectrophotometer (Horiba, Kyoto, Japan), and the slit width was 5 nm.

### 2.8. Photostability of Fluorescence Labeled Silk Fibroin Microparticles

The photostability of the fluorescence-labeled silk fibroin microparticles was examined by inverted fluorescence microscope (TH4-200, Olympus Corporation, Tokyo, Japan). Continuous excitations were performed for 1 min, 5 min, 10 min, 15 min, 20 min, 25 min, and 30 min with blue light (excitation, 495 nm) and green light (excitation, 596 nm), separately, and then, fluorescence values were recorded by the Horiba Fluoromax-4 fluorescence spectrophotometer, and the normalized intensity value was the ratio of every fluorescence value to the maximum fluorescence value.

### 2.9. Cell Culture

The human umbilical vein endothelial cell line EA.hy926 and the human cervical cancer cell line HeLa were purchased from American Type Culture Collection (Manassas, VA, USA). Both cell lines were cultured in Dulbecco’s modified Eagle’s medium (DMEM), supplemented with 10% fetal calf serum (FBS) and 1% antibiotics (penicillin and streptomycin) at 37 °C using a humidified 5% CO_2_ incubator. Under microscopic visualization, cells were seeded when they reached 85%–90% confluence with normal morphology. For studying growth and proliferation, the cells were seeded at a density of 2 × 10^4^ cells/well in a 24-well tissue culture plate (TCP, Corning Inc., New York, NY, USA) with DMEM culture medium (10% FBS and 1% antibiotics) for 24 h.

#### 2.9.1. Cell Proliferation In Vitro

Cell viability tests were carried out using a cell-counting kit-8 (CCK-8) assay. After 24 h of cell seeding at the required density (2 × 10^4^ cells/well) in 24-well plates, the DMEM medium was replaced by serum-free medium which contained SFMPs and SFNPs at different concentrations (0.05, 0.1, 0.3, 0.5, 0.7, and 0.9 mg/mL) and incubated 24 h. After 24 h of incubation with silk fibroin particles, the serum-free medium was removed and replaced with 300 μL of fresh DMEM and 30 μL of the CCK-8 solution for 2 h. Next, the solution was centrifuged at 15,000 rpm for 5 min and the supernatant was placed into 96-well plates at 100 μL/well. The absorbance was recorded at 450 nm in a 96-well plate using a Synergy HT Microplate Reader (Bio-Tek Instruments, Winooski, Vermonts, USA). The cells without incubated particles were used as a control. Cell viability was calculated from a relation of absorbance, i.e., (*A*_sample_/*A*_control_)*100 and plotted as a percentage of viability. *A*_sample_ and *A*_control_ represented the absorbance of the sample and control group at 450 nm, respectively. Each experiment was performed in triplicate and repeated at least three times.

#### 2.9.2. Particles Cultured with Cells

EA.hy926 cells and HeLa cells with a seeding density of 2 × 10^4^ were seeded in 24 well plates and cultured for 24 h. Then, the DMEM medium was replaced by serum-free medium containing 0.5 mg/mL QD-SFMPs and incubated 24 h. At predetermined time intervals (2 h, 6 h, 12 h, and 24 h), fluorescence images from QD-labeled samples were obtained using inverted fluorescence microscope. Excitation wavelength was 596 nm. The co-cultivation process of QD-SFNPs and cells is the same as QD-SFMPs.

### 2.10. Statistical Analysis

All data are listed as the mean ± standard deviation. Statistical analysis was performed using one-way analysis of variance (t-test). The differences at *p* < 0.05 are considered statistically significant.

## 3. Results

### 3.1. Characterization of QD-SFMPs and QD-SFNPs

#### 3.1.1. Morphology 

**Figure 2:**
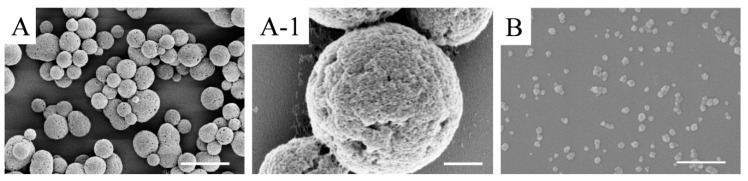
SEM images of silk fibroin particles. (**A**) silk fibroin microparticles (SFMPs); (**A-1**) magnified image of image (**A**); (**B**) silk fibroin nanoparticles (SFNPs). Scale bars: (**A**) 5 μm; (**A-1**) 500 nm; (**B**) 400 nm.

SFMPs and SFNPs were prepared by phase separation and electrospray, respectively. The surface morphology of the particles was observed by SEM as shown in Figure 2. The particle shape of the SFMPs was spherical or ellipsoidal, with a uniform size distribution (Figure 2A), and their surface was rough containing nanometer-sized pores (Figure 2A-1). The pore size was 58.3 ± 20.1 nm. The shape of the SFNPs was irregular and tended to be circular without apparent aggregation (Figure 2B). The average particle diameters of SFMPs and SFNPs calculated using nanometer analysis software from SEM were 1.9 ± 0.4 μm and 51.5 ± 11.0 nm, respectively. Moreover, the sizes of wet particles determined via DLS were 2.7 ± 0.3 μm and 174.8 ± 12.5 nm, respectively. It was obvious that the dry nanoparticles were smaller than those measured by DLS owing to the excellent swelling property of silk fibroin in water.

#### 3.1.2. Principle of Labeling 

**Figure 3:**
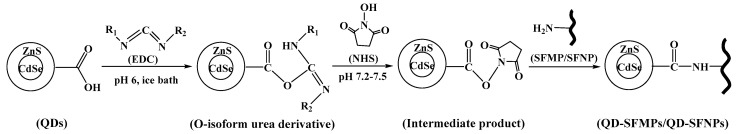
Schematic illustration of Quantum dot (QD)-labeled silk fibroin particles.

CdSe/Zns QDs were modified with polyacrylic acid, resulting in the formation of carboxyl groups on the QD surface. As shown in Figure 3, the carboxyl groups on QDs could be activated by carbodiimide (EDC) to form an O-isoformyl urea derivative as an intermediate product. Then, under the action of N-hydroxysuccinimide (NHS), the unstable intermediate product formed a stable substance which can subsequently react with primary amine groups on the surface of silk fibroin, forming new amide bonds, and the silk fibroin particles were thereby labeled with QDs [39].

#### 3.1.3. FTIR 

**Figure 4:**
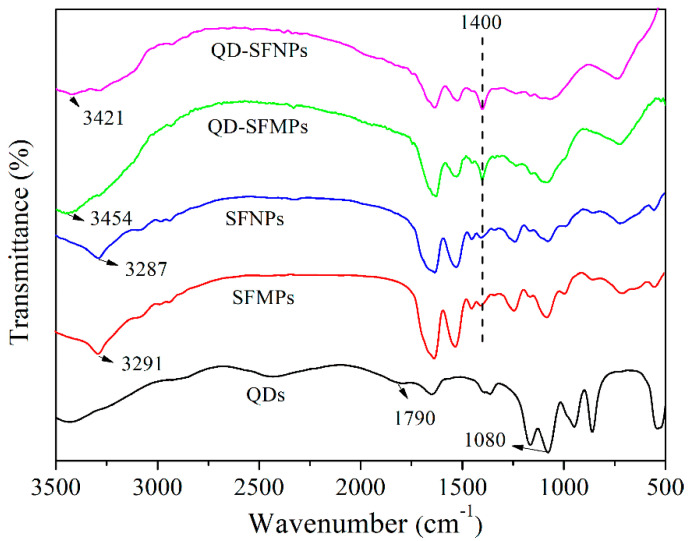
FTIR spectra of silk fibroin particles before and after QD labeling.

Figure 4 showed the FTIR spectra of QDs, SFMPs, SFNPs, QD-SFMPs, and QD-SFNPs. In QDs, there was a strong absorption at 1080 cm^−1^ representing the stretching vibrations of C–O and a weak absorption at 1790 cm^−1^ assigned to the stretching vibrations of COO^–^ [40]. SFMPs showed absorption at 3291 cm^−1^ and SFNPs showed absorption at 3287 cm^−1^, and both represented the stretching vibrations of –NH [41,42]. These particles have an absorption band at 1400 cm^−1^ representing C–N stretching vibration [43]. The spectrum of the QD-SFMPs was different from SFMPs. The absorption at 3291 cm^−1^ was shifted to 3454 cm^−1^ in the QD-SFMPs. Compared with the SFNPs, the absorption at 3287 cm^−1^ was shifted to 3421 cm^−1^ in the QD-SFNPs. Moreover, the intensity of the bands at 1400 cm^−1^ increased in QD-SFMPs and QD-SFNPs. The results indicated that the –COOH groups of the QDs reacted with the –NH_2_ groups of the SFMPs and SFNPs to form new amide bonds. 

#### 3.1.4. Fluorescence Spectroscopy 

**Figure 5:**
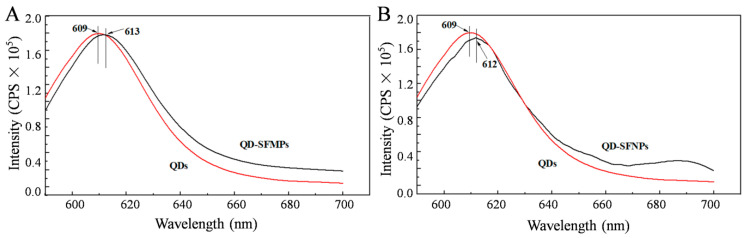
Fluorescence spectra of QDs, QD-SFMPs and QD-SFNPs. (**A**) QDs and QD-SFMPs; (**B**) QDs and QD-SFNPs. Excitation: 550 nm, Emission: 590–700 nm.

Figure 5A,B present fluorescent spectra of the QD-SFMPs and QD-SFNPs, respectively. It can be seen from Figure 5A that the emission peak of the QD solution is 609 nm, and the emission peak of the QD-SFMPs is redshifted by 4 nm to 613 nm, indicating the formation of a new amide bond. In Figure 5B, the 3 nm redshifted fluorescence spectra of the QD-SFNPs also confirm the reaction between the quantum dots and SFNPs. The fluorescence spectroscopy findings are consistent with the results of FTIR spectra.

#### 3.1.5. Fluorescence Stability

**Figure 6:**
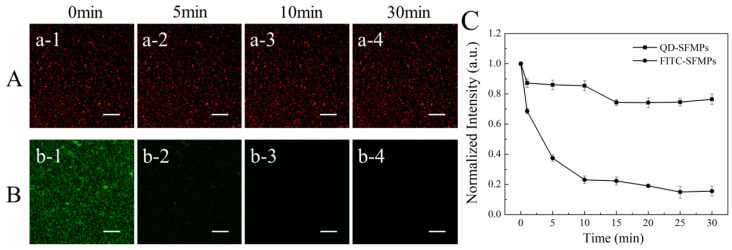
Fluorescence images of (**A**) QD-SFMPs and (**B**) FITC-SFMPs at different time intervals. (**C**) The normalized fluorescence intensity of QD-SFMPs and FITC-SFMPs from 0 min to 30 min, and 1, 2, 3, and 4 denote the irradiation time were 0 min, 5 min, 10 min, and 30 min, respectively. Scale bar: 100 μm.

The fluorescence stability of the silk fibroin particles labeled with QDs was further investigated. The QD-SFMPs and FITC-SFMPs were subjected to continuous laser irradiation under the selected field of view. Figure 6A showed the fluorescence images of QD-SFMPs after continuous irradiation for 30 min. It can be seen that the QD-SFMPs showed high red fluorescence at the initial state (Figure 6Aa-1). As the laser irradiation time increased to 10min, the red fluorescence of the QD-SFMPs slightly decreased (Figure 6Aa-2,a-3), and the red fluorescence remained stable at 30 min (Figure 6Aa-4). The green fluorescence images of the FITC-SFMPs were shown in Figure 6B. The green fluorescence was bright at first (Figure 6Bb-1), but rapidly bleached after 5 min (Figure 6Bb-2). As time increased, the green fluorescence decreased and was almost quenched at 30 min (Figure 6Bb-3,b-4).

The normalized fluorescence intensities of QD-SFMPs and FITC-SFMPs were shown in Figure 6C. During the first 1 min of excitation, the normalized fluorescence intensity of the QD-SFMPs decreased to 87.2%. At 5 and 10 min, the normalized intensities were 86.7% and 85.4%, respectively. The normalized intensity slightly decreased to 74.3% at 15 min and no obvious changes were observed after 15 min, indicating that the intensity of the QD signal tended to stabilize. However, during the first 1 min of irradiation with the FITC-SFMPs, the normalized intensity of FITC decreased to 68.6%, while at 5 and 10 min, the normalized intensity was 37.5% and 23.1%, respectively, indicating a sharp decline in intensity with continued excitation. From 15 to 30 min, the normalized intensity dropped from 22.3% to 15.5%, showing weak fluorescence. The results indicated that the QDs have high stability, excellent resistance to photobleaching and a long fluorescence lifespan, and thus are superior to FITC.

### 3.2. Cell Proliferation 

**Figure 7:**
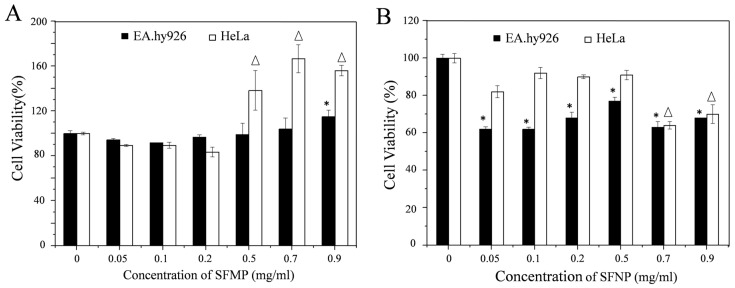
EA.hy926 and HeLa cell viability after 24 h of incubation with different concentrations of SFMPs and SFNPs. blank cell as a control. (**A**) SFMPs; (**B**) SFNPs. * and Δ data points for the experimental group that were statistically significant compared with the control group (*p* < 0.05).

As shown in Figure 7A, the cell viability of EA.hy926 and HeLa cells cultured with different concentrations of SFMPs was investigated by CCK-8 assay. When the concentration of SFMPs was <0.2 mg/mL, the EA.hy926 and HeLa cell viabilities were above 80%, indicating that the SFMPs showed negligible effects on EA.hy926 and HeLa cell proliferation. When the concentration of SFMPs was >0.5 mg/mL, the cell viability of HeLa significantly improved and reached a maximum at 0.7 mg/mL; moreover, the cell viability of EA.hy926 cells increased at 0.9 mg/mL, suggesting that SFMPs are beneficial for the proliferation of EA.hy926 and HeLa cells. Figure 7B showed the cell viability of EA.hy926 and HeLa cells cultured with different concentrations of SFNPs. The cell viability of EA.hy926 cells was significantly lower than that of the blank group (*p* < 0.05), indicating the ability of SFNPs to inhibit EA.hy926 cell growth. The cell viability of HeLa cells was not obviously different from that of the blank group when the concentration of SFNPs was <0.5 mg/mL, but was clearly lower when the concentration was >0.7 mg/mL (*p* < 0.05), illustrating that SFNPs also inhibited HeLa cell proliferation.

Comparing Figure 7A,B, it was found that different-sized particles showed different cell proliferation rates for the same cells. For EA.hy926 cells, the cell viability was above 90% after adding SFMPs, and when the concentration increased to 0.9 mg/mL the cell viability increased. However, when SFNPs were added, the cell viability significantly decreased compared to that of the control (*p* < 0.05). For HeLa cells, the cell viability was above 80% after adding SFMPs then significantly increased at high concentrations (>0.5 mg/mL). However, cell proliferation was inhibited after adding SFNPs and then significantly decreased when the concentration was >0.7 mg/mL. Further, we tested the cell proliferation of EA.hy926 cells when cultured in serum-free medium containing 0.5 mg/mL QD-SFNPs for 24 h. The cell viabilities of EA.hy926 was 77.5 ± 1.9% and 69.9 ± 1.6% when cells cultured with SFNPs and QD-SFNPs, respectively, indicating that QD-labeled SFNPs had toxicity to the cells. However, the cell proliferation was about 70% which did not affect the tracking observation of the QD-labeled particles in our study.

### 3.3. Observation of Silk Fibroin Particles Cultured with Cells

**Figure 8:**
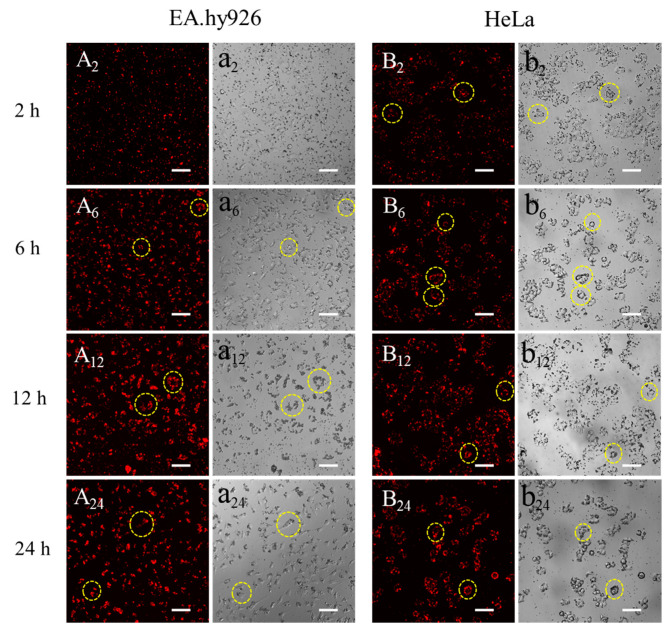
Fluorescent images of QD-SFMPs cultured with (**A**,**a**) EA.hy926 cells and (**B**,**b**) HeLa cells. A and B denote the fluorescent field, while a and b denote the bright field, and 2, 6, 12, and 24 denote incubation time was 2 h, 6 h, 12 h, and 24 h, respectively. Scale bar: 100 μm. The yellow circles indicated that the QD-SFMPs likely tended to adhere to the cell surface.

To investigate the interactions between microparticles and different cell lines, SFMPs labeled by QDs were incubated with EA.hy926 and HeLa cells at a concentration of 0.5 mg/mL for 24 h (Figure 8). For EA.hy926 cells, most of the QD-SFMPs were free around the cells after incubation for 2 h (Figure 8a_2_). At this time, the red fluorescence was uniformly dispersed in the plane (Figure 8A_2_). After 6 h of culture, partial QD-SFMPs were observed on the cell surface (Figure 8a_6_, seen in the yellow circle symbols) accompanied with the accumulation of fluorescence on the cell surface (Figure 8A_6_), indicating that the cells were adhered onto the QD-SFMPs. As the incubation time extended, more QD-SFMPs were located on the cell surface and there were few free QD-SFMPs in the plane at 24 h (Figure 8a_12_,a_24_). The fluorescence aggregated on the cell edge after culturing for 24 h (Figure 8A_12_,A_24_), suggesting that most QD-SFMPs tended to adhere to the EA.hy926 cell surface. HeLa cells behaved similarly to EA.hy926 cells. QD-SFMPs emerged on the cell surface at 2 h (Figure 8b_2_, seen in the yellow circle symbols) and the fluorescence showed a tendency to aggregate (Figure 8B_2_), indicating that the cells might be adhered to the QD-SFMPs at this time. When the culture time was increased to 12 h, more QD-SFMPs were located on the cell surface (Figure 8b_2_,b_6_,b_12_) along with the local fluorescence intensity increased (Figure 8B_2_,B_6_,B_12_). After 24 h of culture, the fluorescence mainly emerged in the cell edge with no significant change in the intensity compared with 12 h (Figure 8B_24_), suggesting that most QD-SFMPs were likely to adhere to the HeLa cell surface. In group a, the EA.hy926 cells were spindle-shaped within 24 h, and there was also no obvious change in the morphology of HeLa cells within 24 h in group b, indicating QD-SFMPs had no significant effects on cell morphology. 

**Figure 9:**
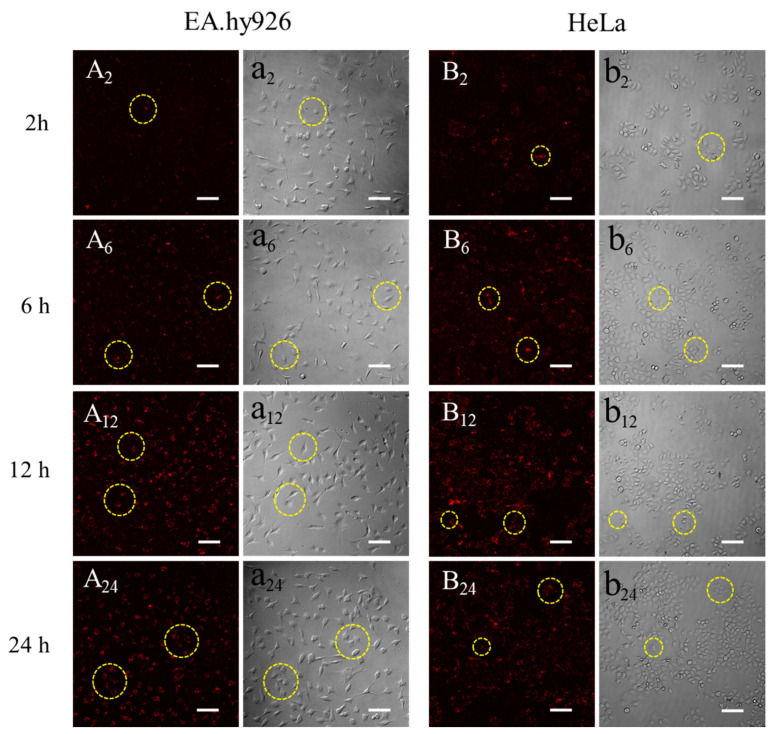
Fluorescent images of QD-SFNPs when cultured with (**A**,**a**) EA.hy926 cells and (**B**,**b**) HeLa cells. A and B denote the fluorescent field while a and b denote the bright field, and 2, 6, 12, and 24 denote incubation time was 2 h, 6 h, 12 h, and 24 h, respectively. Scale bar: 100 μm. The yellow circles indicated that the QD-SFNPs were more likely to be internalized by the cells.

Figure 9 showed the fluorescent images of 0.5 mg/mL QD-SFNPs co-incubated with EA.hy926 and HeLa cells. The EA.hy926 cells were spindle–shaped (Figure 9a_2_) and the weak fluorescence was observed in cells at 2 h (Figure 9A_2_, seen in the circle symbols), this may indicate the possibility of QD-SFNPs internalization by EA.hy926 cells. With the extension of culture time, it seemed that more QD-SFNPs were internalized by EA.hy 926 cells as the fluorescence intensity in cells increased (Figure 9A_6_,A_12_). At 24 h, the fluorescence intensity did not change significantly (Figure 9A_24_), and it looked like that EA.hy926 cells have internalized most of the QD-SFNPs. The interaction of HeLa cells with QD-SFNPs was similar to EA.hy926 cells. Red fluorescence was observed in cells at 2 h (Figure 9B_2_, seen in the circle symbols), indicating that QD-SFNPs might have been internalized by HeLa cells at this time. As time elapsed, the fluorescence intensity in the cells was enhanced (Figure 9B_6_,B_12_), showing a possible increase of internalized QD-SFNPs. At 24 h (Figure 9B_24_), there was no obvious change in fluorescence intensity compared with 12 h, and it seemed that most QD-SFNPs tended to be internalized by HeLa cells. From the bright field images, the EA.hy926 cells remained spindle–shaped within 24 h (Figure 9a_2_,a_6_,a_12_,a_24_), suggesting that internalized QD-SFNPs do not affect cell morphology significantly. For HeLa cells, the morphology of most cells was oval within 24 h. However, several round cells were observed at 2 h (Figure 9b_2_), and the number of round cells slightly increased with the time extended to 24 h (Figure 9b_6_,b_12_,b_24_), indicating that internalized nanoparticles might induce HeLa cell apoptosis. 

The results from Figure 8 indicated that QD-SFMPs likely tended to adhere to EA.hy926 and HeLa cell surface. Figure 9 suggested that QD-SFNPs were more possibly internalized by EA.hy926 and HeLa cells. They all showed that the interactions between particles and cells occurred within 12 h; in view of this, our observation time lasted 24 h. 

## 4. Discussion

One of the major challenges of current micro/nanoparticles-mediated drug delivery is delivering drugs to specific sites of action, such as the extracellular or intracellular environment or even specific organelles [44]. The size of particles affects the interactions between particles and cells and, thus, the sites of drug release. Therefore, the interactions of different sizes and types of cells are crucial for drug delivery. QDs as tracers could be easily used to decipher cellular events [27]. In this study, QD-labeled SFMPs and SFNPs were prepared to clarify cellular behavior. However, drugs work differently on normal cells and cancer cells. Human umbilical vein endothelial cells EA.hy926 and cervical cancer cells HeLa were selected.

Silk fibroin particles have been utilized as promising drug delivery materials due to their biocompatibility, degradability, and ability to control drug release kinetics [31]. The silk fibroin particles prepared by salting out method and high-voltage electrospray method exhibited stable *β*-sheet structure, which stabilized the structure of the particles in the aqueous medium system (DMEM and PBS) [16,45]. In this study, regular SFMPs were prepared by salting out, and when silk fibroin precipitated to nucleate at 4 °C, the small precipitated phosphate crystals were embedded, which were further re-dissolved after incubation at room temperature. Consequently, the outer part of the microparticles exhibited nanometer-sized pores (Figure 2A-1). High-voltage electrostatic-based techniques (Figure 1) were selected to prepare SFNPs due to the advantages, including a controllable particle shape, surface morphology, and particle size, as well as the production of particles with good dispersibility [46]. Due to the electric potential between the needle and the receiving plate, the surface of the silk fibroin solution will accumulate a high-density charge, which will break up into a series of small droplets under the action of the electric field force to overcome the surface tension. The droplets are not differentiated completely and microspheres with uniform particle size cannot be formed at low voltage. Excessively high voltage will make the Taylor cone extremely unstable and have poor particle size uniformity. The previous experimental results show that when the voltage is controlled at 7–11 kV, it can be continuously and uniformly differentiated, and the particle size can be controlled [16]. The SFNPs were shown in Figure 2B. The wet diameters were also measured by DLS for the micro/nanoparticle transport drugs in the liquid human microenvironment. The diameters of particles under wet conditions were larger than those dry conditions because silk fibroin has excellent swelling properties in water [47].

To track the location of silk fibroin particles in the cell, QDs modified by –COOH groups were selected. The amino groups of silk fibroin could be conjugated with the carboxyl groups of QDs under the mediation of EDC/NHS [23,39]. The mechanism was shown in Figure 3. The FTIR provided evidence for the synthesis of QD-SFMPs and QD-SFNPs (Figure 4). In QDs, with the formation of new amide bonds, the O adjacent to C was replaced by N which has a lower electronegativity. Therefore, the electron cloud of the polymer becomes dispersed along with the decrease of the system energy, causing the weak redshifts of the fluorescent peaks in Figure 5 [48]. For particles labeled with fluorophores, and the fluorescence stability plays an important role ensuring the validity and accuracy of the in vitro assessment. FITC showed the highest fluorescent signal at the excitation wavelength of 495 nm [49] while QDs showed the strongest fluorescence at the excitation wavelength of 596 nm [50]. Therefore, 495 nm and 596 nm as the excitation wavelengths of FITC-SFMPs and QD-SFMPs were selected, respectively, to achieve their strongest fluorescence and the fluorescence stability was compared under their own strongest fluorescence. Core-shell QDs have a high fluorescence stability because the shell saturates defect states and dangling bonds at the core surface, which otherwise favor the undesired nonradiative recombination of the photogenerated electron–hole pairs and long-wavelength emission and, therefore, significantly increase the luminescence quantum yield, resulting in photostable fluorescent probes that are superior to conventional dyes [24]. Therefore, the fluorescence stability of QDs was superior than FITC as the results showed that the fluorescence stability of QD-SFMPs maintained 70% of the original value and remained stable after continuous irradiation for 30 min while the green fluorescence of FITC-SFMPs rapidly bleached after 5 min (Figure 6).

Cell proliferation assays are used for carrier screening to detect whether the particles have effects on cell proliferation or display direct cytotoxic effects [16]. The large surface area of SFMPs offered adequate space for cells to adhere, spread, and proliferate. The pores of SFMPs are also conductive to the precipitation of the extracellular matrix and cellular adhesion, growth, and cell proliferation can be supported [37]. Therefore, the proliferation of EA.hy926 and HeLa cells was improved after being cultured with 0.9 mg/mL SFMPs (Figure 7A). Compared with EA.hy926, HeLa cells have a stronger adherence at high concentration (>0.5 mg/mL) which results in the HeLa cell proliferation significantly improving [51]. In contrast to those with SFMPs, the cell viabilities of EA.hy926 and HeLa cells decreased in the presence of SFNPs (Figure 7B); we think this was mainly due to the nanoparticles possessing a high surface area, which increased the chance of interacting with surrounding biomolecules and, as a consequence, triggered an adverse response [52]. At a low concentration of SFNPs (0.05 mg/mL), the EA.hy926 cell proliferation was significantly inhibited. The results were similar to the previous studies where the nanoparticles with concentration of <240 μg/mL were significantly cytotoxic for vascular endothelial cells [53]. Interestingly, though the normal cell proliferation was significantly inhibited when the concentration of SFNPs < 0.5 mg/mL, there is tendency that the cell viability is increased. It could be possible that some SFNPs aggregated at higher concentration which prevented the nanoparticles entering the cells because of the increasing size and consequently the cell viability increased [54]. The effect of nanoparticles on cell viability is a complex process with multiple mechanisms, and more evaluations are necessary in the future. 

Adhesion is the first step between particles and cells [6]. Cells can be adhered to the surface of silk fibroin micro/nanoparticles after the biological information carried by amino sequences is recognized [1]. When the adhesive properties were ascertained, the cell extended filopodia to guide cell migration [55], which caused more particles to be recognized and adhere to the cell surface. The surface zeta potentials of SFMPs and SFNPs were both around −11.8 mV, indicating that there was a large amount of negative charge on the surface of the particles, and the electrostatic repulsion between the particles prevented the nanoparticles from aggregation in the serum-free medium. In our study, we observed that QD-SFMPs were present on the EA.hy926 and HeLa cell surface at 6 h and 2 h, respectively (Figure 8A_6_,a_6_,B_2_,b_2_, seen in the yellow circle symbols), indicating the adhesive interactions between cells and microparticles have occurred. With time extended to 12 h, more particles emerged on the cell surface, resulting in the accumulation of fluorescence. However, the fluorescence intensity existed in the cell edge did not change significantly at 24 h (Figure 8A_12_,A_24_,B_12_,B_24_), suggesting that QD-SFMPs mainly tended to adhere to the cell surface. This phenomenon might be due to the fact that the particles entering the cell have a certain critical radius that is less than the large size of microparticles, causing microparticles to be excluded from the cell membrane [7]. The results were similar to those of Nandana [56], who reported that mouse fibroblast cells L929 were adhered to the surface of SFMPs with diameters of 4–5μm. In contrast, cells internalize nanoparticles, which involves a binding (adsorbing) process followed by the formation of vesicles, and then, the formed nanoparticle-containing vesicles are internalized by endocytosis [11]. It has been reported that a nanoparticle size of ~50 nm may result in higher tumor cell uptake and mesoporous silica nanoparticles ~50 nm in diameter had the highest loading in HeLa cells [57,58]. In our study, ~50 nm QD-SFNPs (~170 nm in wet state) showed the possibility of being internalized by EA.hy926 and HeLa cells (Figure 9, seen in the circle symbols). However, the shape of EA.hy926 remained spindly after internalizing QD-SFNPs within 24 h, while local Hella cells became round; this might be due to the fact that nanoparticles taken up by HeLa cells induced cellular and mitochondrial dysfunction [59]. Therefore, SFNPs are potential carriers for tumor cells. 

For normal cells, such as human umbilical vein endothelial cells EA.hy926, delivering bioactive drugs to cells is needed to speed up cell proliferation to facilitate tissue repair. SFMPs encapsulating insulin have been applied [60]. The released insulin from those SFMPs binds to receptors on the cell surface and then shows a strong stimulatory effect on the migration of endothelial cells, which would be positive for vascularization in the wound healing process. Consequently, when delivering drugs that act on normal cell membranes, SFMPs are more likely to be preferred. For cancer cells, there are drug efflux proteins on the cell membrane. One example is membrane-bound p-glycoprotein (P-gp), which reduces the intracellular concentration of cancer cells [61]. P-gp may also be present on the nuclear membrane, limiting the transport of drugs into the nucleus [62]. Therefore, delivering drugs to the inside of the cancer cell is necessary. SFNPs might be internalized by HeLa cells and inhibit cell proliferation, which might be useful for nanoparticles delivering drugs to kill or inhibit cancer cells. Although the potential mechanism of internalization and cellular location of SFNPs have yet to be investigated, the results of this paper inspired that SFMPs may be preferred if the drugs need to be delivered to normal cell surface, while SFNPs may be preferred if the drugs need to be delivered to tumor cells, providing a reference for the selection of appropriate size particles as carriers.

## 5. Conclusions

In this paper, quantum dot-labeled silk fibroin particles with favorable fluorescent intensity stability were fabricated to investigate the cellular behavior toward microparticles and nanoparticles. The proliferation results showed that the silk fibroin microparticles promoted EA.hy926 and HeLa cell proliferation, while the silk fibroin nanoparticles inhibited cell proliferation. In vitro, the results of tracing fluorescence-labeled silk particles showed interesting phenomena in that EA.hy926 cells mainly tended to be adhered to the surface of microparticles while HeLa cells were more likely to internalize the nanoparticles, which suggested that the particle size affects the cell responses when particles are co-cultured with cells. The results indicated a selection in terms of particles size that microparticles might be beneficial to deliver drugs on normal cell surface, while nanoparticles may be preferred to deliver drugs inside cancer cells.
